# Propagation of human germ stem cells in long-term culture

**Published:** 2013-07

**Authors:** Mohammad Mehdi Akhondi, Arash Mohazzab, Mahmood Jeddi-Tehrani, Mohammad Reza Sadeghi, Akram Eidi, Abbas Khodadadi, Zeinab Piravar

**Affiliations:** 1 *Reproductive Biotechnology Research Center, Avicenna Research Institute, ACECR, Tehran, Iran. *; 2 *Monoclonal Antibody Research Center, Avicenna Research Institute, ACECR, Tehran, Iran. *; 3 *Department of Biology, Science and Research Branch, Islamic Azad University, Tehran, Iran. *; 4 *Research and Preparation Center, Iranian Tissue Bank, Tehran University of Medical Science, Tehran, Iran.*

## Abstract

**Background:** Spermatogonial stem cells (SSCs), a subset of undifferentiated type A spermatogonia, are the foundation of complex process of spermatogenesis and could be propagated in vitro culture conditions for long time for germ cell transplantation and fertility preservation.

**Objective:** The aim of this study was in vitro propagation of human spermatogonial stem cells (SSCs) and improvement of presence of human Germ Stem Cells (hGSCs) were assessed by specific markers POU domain, class 5, transcription factor 1 (POU5F1), also known as Octamer-binding transcription factor 4 (Oct-4) and PLZF (Promyelocytic leukaemia zinc finger protein).

**Materials and Methods:** Human testicular cells were isolated by enzymatic digestion (Collagenase IV and Trypsin). Germ cells were cultured in Stem-Pro 34 media supplemented by growth factors such as glial cell line-derived neurotrophic factor, basic fibroblast growth factor, epidermal growth factor and leukemia inhibitory factor to support self-renewal divisions. Germline stem cell clusters were passaged and expanded every week. Immunofluorecent study was accomplished by Anti-Oct4 antibody through the culture. The spermatogonial stem cells genes expression, PLZF, was studied in testis tissue and germ stem cells entire the culture.

**Results:** hGSCs clusters from a brain dead patient developed in testicular cell culture and then cultured and propagated up to 6 weeks. During the culture Oct4 were a specific marker for identification of hGSCs in testis tissue. Expression of PLZF was applied on RNA level in germ stem cells.

**Conclusion:** hGSCs indicated by SSCs specific marker can be cultured and propagated for long-term in vitro conditions.

This article extracted from Ph.D. Thesis. (Zeinab Piravar)

## Introduction

Spermatogenesis is a complex process originates from Spermatogonial stem cells (SSCs) that is maintained through adult life of the male testes. SSCs that are a subset of undifferentiated type A spermatogonia, place in a stem cell niche at the basement membrane (BM) of the seminiferous tubules (1). Spermatogenesis begins at 5-7 days post natal in rodents and 10-13 years after birth in men (2). Previous experiments on rodent spermatogonia demonstrated efficiently isolation and short or long-time culture of these cells (3-5). According to these studies, factors that control the fate determination of the cells and regulation of early phases of spermatogenesis by molecular mechanisms are considered. However, there are many restrictions on the research on human spermatogonia.

One reason for such a little advance in human spermatogonia and SSCs studies has been limited amounts of normal human testis for research purposes (6). Another reason has been restricted cell divisions and consequently close distance between undifferentiated and differentiated spermatogonia during spermatogenesis process compare with rodents and other primates (7). Clermont in 1963 characterized two types of undifferentiated spermatogonia in human testis; the A_dark_ and A_pale_ spermatogonia that are different in their chromatin distribution. They informed that the A_dark _was reserve stem cells while the A_pale _was renewing stem cells and both of them were placing in stem cell niche at the basement membrane of seminiferous tubules (1, 7-9). However, after more than forty years of Clermont’s findings, very little new progression is available on the identification, renewing or differentiation of human SSCs (10). 

Specific growth factors were examined in extra cellular matrix or niche of SSCs stimulate self-renewing and maintenance of these cells as glial cell line-derived neurotrophic factor (GDNF) that produce by sertoli cells. According to this observation, a long-term SSC culture was created mentally in which SSCs self-renewing and proliferation was promoted in the support of GDNF, Epidermal growth factor (EGF) or basic fibroblast growth factor (FGF2) and presence of fetal bovine serum (FBS) (11).

In our study, specific markers have been identified for SSCs and progenitors in other species to distinguish hGSCs phenotypes. POU domain, class 5, transcription factor 1 (POU5F1), also known as Octamer-binding transcription factor 4 (Oct -4), a nuclear marker for SSCs and progenitor cells expresses in many species such as mouse, rat and monkey. 16 Promyelocytic leukemia zinc finger protein [PLZF, also known as zinc finger and BTB domain containing 16 (ZBTB16)] is a nuclear spermatogonial-specific marker that is well-known in many species (3, 4, 6, 12-15). 

The development of the spermatogonial transplantation technique for therapeutic agents, has given new impetus to research on SSCs (16, 17). Because testicular tissues do not contain sufficient SSCs to fully colonize the testis after transplantation, in vitro propagation of hGSCs will be necessary to obtain an adequate amount of cells for successful transplantation. Such culture methods have been recently developed in animal model systems but have thus far not been reported for hGSCs (11-14). We demonstrated here on an in vitro culture system that allows for long-term culture and propagation of human spermatogonial stem cells and identified these cells by specific markers, OCT-4 and PLZF.

## Materials and methods

**Human germ stem cells culture**

In this experimental study, testis samples were donated from a brain dead 44 years old patient with acquisition consent inform. Ethical aspects of sampling and other procedure of study were approved by Avicenna institute ethics committee and National Ethical Committee on Research in Medical Sciences. Spermatogenesis process was normal in testis tissue. The sample was cut into small pieces (25 mm^3^) and stored by cryopreservation method in 10% dimethyl sulfoxide (DMSO) (Sigma-Aldrich, St Louis, Missouri, USA), 20% FBS (Invitrogen, Grand Island, USA) and 70% Dulbecco's Modified Eagle Medium: Nutrient Mixture F-12^o^ (DMEM/F12) as basal media and at -196^o^C for later applications. 

Frozen testis tissue were defreeze in 37^o^C water bath in 2 min. The tissue was minced by sterile scissors and dispersed by a two-step enzymatic digestion including collagenase (type IV, Sigma-C1889) 5 mg/ml and DNase 1mg/ml for 30 min followed by 0.25% trypsin/1 mM EDTA digestion (both from Invitrogen) for 10 min. Approximately 2×10^6^ cells were gained by this procedure per each tissue pieces (25 mm^3^). The number of dead cells was generally less than 5% as assessed by trypanblue staining. 

Testicular cells were cultured in StemPro-34 SFM (Invitrogen) supplemented with StemPro supplement (Invitrogen), 1 nonessential amino acids, 15 mM 4-[2-hydroxyethyl]-1 piperazineethanesulfonic acid [HEPES], 0.12% sodium bicarbonate, 4 mM L-glutamine (all from Invitrogen), penicillin (100 IU/mL) (Sigma), streptomycin (100 μg/mL) (Sigma), fangizone 40 μg/mL, insulin 25 mg/ml, transferrin 100 mg/ml, putrescine 60 mM (ITS, Sigma P6024), sodium selenite 30 nM, D-(1)-glucose 6 mg/ml, pyruvic acid 30 mg/ml, DL-lactic acid 1 ml/ml (Sigma), bovine albumin 5 mg/ml (ICN Biomedical, Irvine, CA,USA), L-glutamine 2mM, 2-mercaptoethanol 5×10^-5^ M, minimal essential medium (MEM) vitamin solution (Invitrogen), MEM nonessential amino acid solution (Invitrogen), ascorbic acid 10^-4^ M, d-biotin 10 mg/ml (Sigma B4639), β-estradiol 30 ng/ml (Sigma B 2257), progesterone 60 ng/ml (Sigma), recombinant human EGF (20 ng/mL) (Sigma-Aldrich), recombinant human GDNF (10 ng/mL) (Sigma-Aldrich-G1777), and 10ng/mL recombinant human Leukemia inhibitory factor (LIF) (Chemicon International Inc., Temecula, California, USA) and 10% FBS. The cells were incubated at 37^o^C in 5% carbon dioxide in air. Germ cells were passaged by short-term trypsination every 10 days at 80-90% confluency to 1 or several new dishes. We used 2% FBS after 14 days of starting culture to prevent overgrowth of fibroblasts. 

**Immunohistochemistry**

Immunohistochemistry by using anti human Oct4 (Clone 3B8 B12, Avicenna Research Institute, Tehran, Iran) as primary antibody was performed. To prepare tissue sections, fresh human tissues were embedded in OCT compound in cryomolds and frozen in liquid nitrogen. Frozen blocks were stored at -80^o^C. Cryostat sections were cut in 4-8 µm thickness and fixed on slides. Slides were kept at -80^o^C until needed. Before immunostaining, slides warmed at room temperature for 30 minutes and ice cold acetone fixation was done for 5 minutes and washed in phosphate buffered saline (PBS). We prevent non-specific binding by blocking with 10% normal serum. Primary antibodies (AB) incubation including mouse monoclonal AB to Oct-4 (Clone 3B8 B12, Avicenna Research Institute, Tehran, Iran) at a 1: 50 dilution was performed for 90 min. 

After 3 times washing with PBS, testis sections were incubated with secondary antibody to FITC-conjugated anti-mouse IgG (SPH F301, Avicenna Research Institute, Tehran, Iran) at a 1:500 dilution for 45 min. The same washing process was also done. The 4', 6'-Diamidino-2-phenylindole (DAPI) was used to stain the nuclei of the cells and the sections were observed for epifluorescence using an Olympus Fluoview 500 Laser Scanning Microscope (Olympus, Melville, NY, USA). Immunocytochemistry was performed on germ cells after culture for 4 weeks. Slides were made ready with cytospin and following steps are like immunohistochemistry. Replacement of primary antibody with PBS and IgG was used as a negative control.

**Gene expression**

In this study, we applied specificity of PLZF for human germ stem cells to identify these cells. Presence of SSCs clusters during the entire culture was proved by studding the expression of spermatogonial genes (18, 19). Total RNA from cultured testicular cells, sub cultured germline stem cells and testis tissue as a positive control was extracted by RNX^TM^-Plus (CinnaGen Co. Cat No. RN7713C). Simple Reverse transcriptase polymerase chain reaction (RT-PCR) was carried out by first-strand cDNA synthesize with random hexamers and the superscript II preamplification system (Invitrogen). 

Simple RT-PCR reaction was proceeded with specific primers for PLZF (ZBTB16) (Forward: 5' GGTCGAGCTTCCTGATAACG 3', Reverse: 5' CCTGTATGTGAGCGCAGGT 3' product size: 149 bp) with master mix kit (Taq DNA polymerase Master mix RED Amplicon) the annealing temperature (*Ta*) was 60^o^C. GAPDH primer was utilized as DNA improvement (forward: 5' AGAAGCTGGGGC TCATTTG 3', Reverse: 5' AGGGCCATCCAC ACGTCTTC 3' product size: 129)

**Statistical analysis**

Statistical analysis was performed by ANOVA using SPSS statistical software version 2012. Statistically significant differences (p<0.05) were determined between Germ line stem cell clusters in first and last weeks of culture. 

## Results

**Isolation and culture of Human Germ Stem Cells (hGSCs) from human testis tissues**

**Figure 1:**
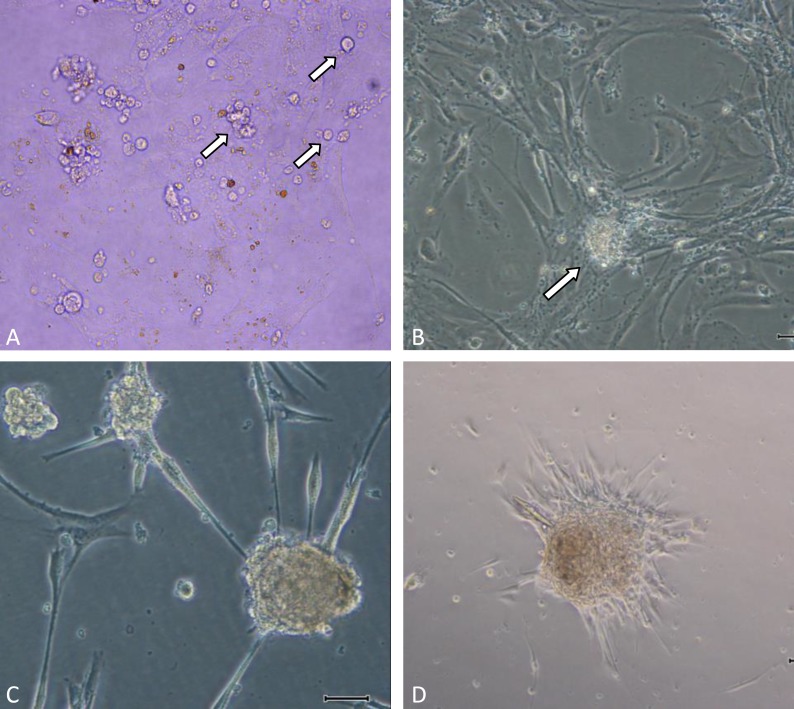
Germline Stem Cell (GSC) Clusters in vitro culture condition.

In the initial attempt to isolate human germ stem cells we obtained testis tissue from a brain dead patient and produced cell suspensions by two step enzymatic digestions. Isolated cells were cultured on plates in StemPro media supplemented with growth factors and 5% FBS to avoid over growth of somatic cells as fibroblasts. However, although the resulting cells were capable of being propagated in vitro, they did not made colonies up to 2-3 weeks of culture initiating. Following enzymatic dissociation of the testis tissue, after approximately 10-14 days of culture, very small colonies as individually clumps of visible cells started to grow on top of the monolayer of testicular somatic cells (Figure 1 A and B). As an alternative, manual passaging of colonies was performed. These colonies could be successfully propagated in vitro with passaging via trypsin digestion. After ten days colonies arose to the original size and cells were trypsinized over again. 

**Figure 2:**
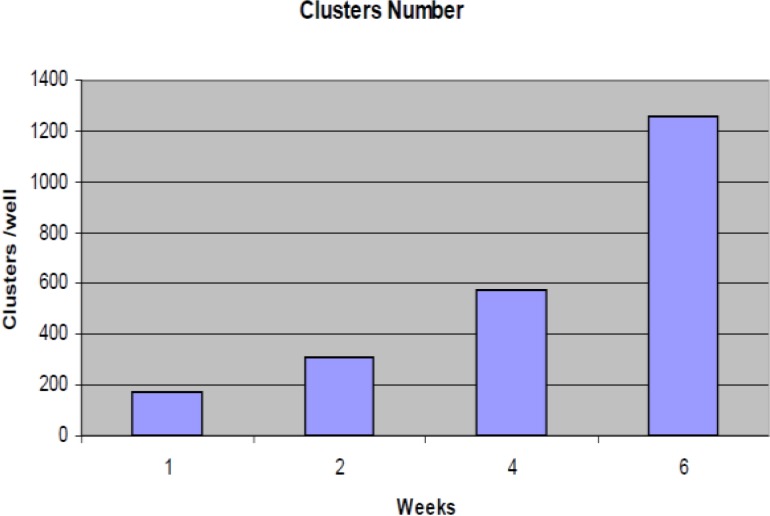
Diagram of Germ line stem cells cluster numbers during 6 weeks culture in a 6 well culture dishes (p<0.05).

These cells, which we have termed human germ stem cells (hGSCs), have been propagated for approximately 6 weeks in vitro presented as colonies with large and round shape (Figure 1 C and D). Statistical analysis presented increasing in germ stem cell cluster numbers from first to 6 weeks of culture (Figure 2)

**Immunostaining analysis**

**Figure 3:**
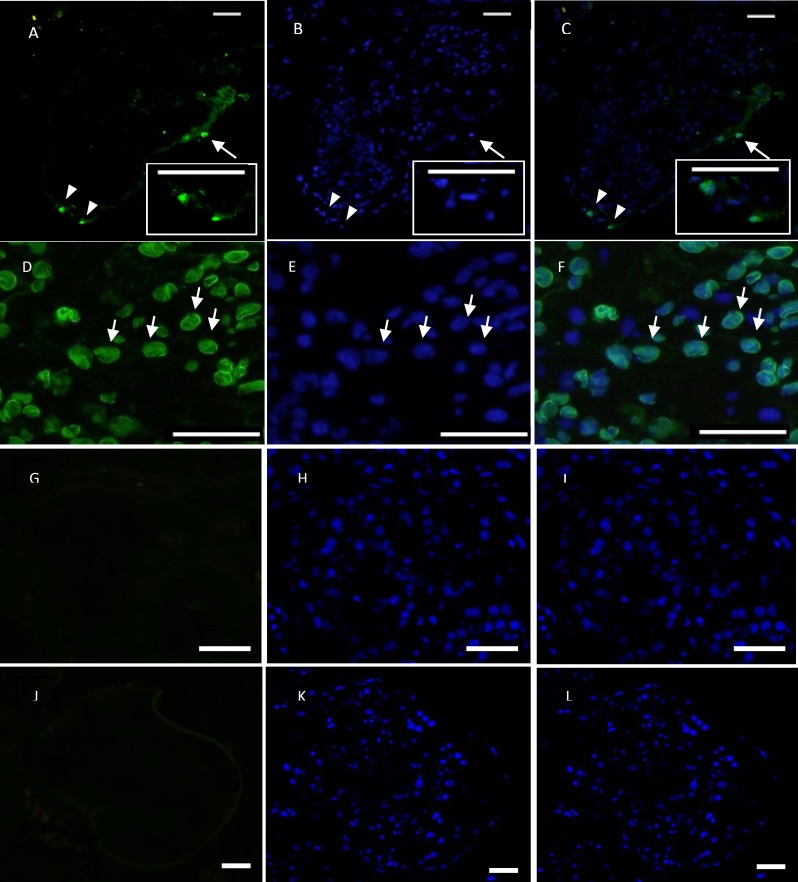
immunofleurescent staining for anti OCT-4 antibody specified for Germline Stem Cells (GSCs).

In this study for spermatogonial stem cell identification, immunohistochemistry was performed using Oct-4 as specific marker of these cells. This technique revealed Oct-4 in a subpopulation of spermatogonial nucleus placed on seminiferous tubules basement membrane in adult testis tissue. We did not determined Oct-4 in sertoli cells or differentiated germ cells. A few spermatogonia were detected for Oct-4 in each seminiferous tubule cross section (Figure 3, A-C). Normal mouse IgG and PBS were used as negative controls. Immunocytochemistry revealed that Oct-4 is present in the nucleus of more than 40% of the isolated human spermatogonia after 6 weeks (Figure 2, D-F).

**RT-PCR analysis**

RT-PCR was performed to confirm the expression of hGSCs specific gene PLZF (Promyelocytic leukemia zinc finger protein) in adult human testis and throughout the entire culture period at passages 3 and 6 relative to a normal human testis sample. GAPDH primer proved cells presentation during RT-PCR process. 

**Figure 4:**
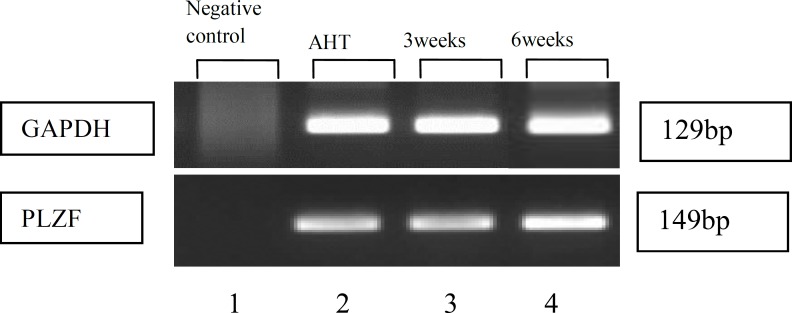
RT-PCR representation for spermatogonial stem cells marker PLZF in adult human testis (AHT) tissue in column 2 and after 3 and 6 weeks, column 3 and 4. GAPDH was used as housekeeping gene. Negative control is presented in column 1

Results demonstrated that the hGSCs at passages 3 and 6 express a specific gene PLZF (Promyelocytic leukemia zinc finger protein) expressed in the testis, as shown in figure 4.

A, Phase contrast image of cultured human testicular cells before colony formation (7 days after digestion).

B, Germ stem cell colonies (arrows) after 2 weeks.

C and D, germ stem cell clusters after 4 and 6 weeks of culture. Scale bar 50um.

A: Human seminiferous tubules cross sections. OCT-4 positive cells or spermatogonial stem cells are in basement membrane of seminiferous tubules. B: DAPI. C: Merge. D-F: Isolated human spermatogonial stem cells in 6weeks of culture. Arrow shows spermatogonial stem cell. E: DAPI. F: Merge. Arrow heads locate spermatogonial stem cells in seminiferous tubules on basement membrane that was magnified. G: Human testis tissue, Negative control by PBS. H: DAPI. I: Merge. J: Human testis tissue, Negative control by IgG. K: DAPI. L: Merge. Scale bar: 50µm.

## Discussion

In this study, isolation, culture and identification of human undifferentiated spermatogonia allows us to characterize these putative cells in human testes phenotypically. We used basal media that formulated to support the development of human hematopoietic stem cells but later used to culture spermatogonial stem cells in other species. In our culture condition human spermatogonial stem cells increased in number by self-renewal in vitro. Growth factors that we used in this culture medium included GDNF, bFGF, EGF, and LIF that were able to promote spermatogonial stem cell survival and proliferation (11). 

In this culture condition, germ stem cell (GSCs) clusters presented as clumps of individually visible cells after 2-4 weeks of culture initiation. We could characterized them by expression of markers as Oct4 and PLZF that had been proved as germ stem cell markers in many species including human (14, 18, 19). In addition to clusters that presented morphologically and phenotypically being GSCs, we observed another shape of colonies with compact and sharp edge that were resemble to colonies that called embryonic like stem (ES-like) cells in other studies (20). 

ES-like cells suggested to be pluripotent and able to convert to all kind of cells of tree germinal layers. It suggested that ES-like cells express embryonic stem cell markers but they were negative for PLZF that proved as GSCs marker (21). In contrast to most animal studies and recent human studies (5, 19). We did not eliminate residual testicular somatic cells from the cell suspension and used them as feeder cells, because these somatic cells are capable of supporting spermatogonial cells in culture. We successed to prevent overgrowthing of adult human somatic cells during the culture by adding low concentration of FBS (2-5%). 

So, GSCs co-culture with somatic cells was not inconvenience. Additionally, serial passaging motived declination of these cells after 6 weeks and they could not support cluster of GSCs as feeder layer. These events are compatible with Sadri-Ardekani *et al* results (19). There were two strategies to continue the culture according to previous studies. First, colonies could be transferred to plates that coated with mytomycin C-inactivated somatic cells as feeder layer by allo or autogenic references by non-enzymatic passaging. So, GSCs could be maintained and proliferated for a long time. Seandle *et al* suggested that co-culture of GSCs with somatic cells cause induction of multipotency in GSCs by somatic cells after three months which can form functional differentiated tissues (13). Second strategy is to culture GSCs in feeder free plates or laminin-coated plates. Laminin is secreted by sertoli cells as an adhesion molecule and bind to ITGα6/β1 receptors on the surface of spermatogonial cells (5). 

Therefore, laminin application in feeder free culture could perform best condition for maintenance and proliferation of GSCs for a long time without change in stem cell potency (19). However, according to previous studies epigenetic modification may happen after a long-term culture of mouse SSCs (22). Although this finding has not been studied in human GSCs. Oct-4 has identified the marker of stem cells which can bind to other transcription factors such as Sox and FoxD3 or to the promoter or enhancer regions of many downstream target genes. This gene regulates downstream gene expression in order to maintain the self-renewal of stem cells positively or negatively (23). Oct-4 expression is exclusively positive in SSCs of fetal testicular tissue, but negative when differentiated in type B proliferative spermatogonium. Kubota *et al* reported that SSCs expressed Oct-4 strongly by using a green fluorescent conjugated antibody for labeling (4). They suggested Oct-4 as a useful marker for SSCs. 

In our study, green fluorescent secondary antibody was used for indirect immunofluorescent. Under a fluorescent microscope, these cells were round and consistent with the shape of SSCs with nucleus staining. Little nonspecific staining could be observed that confirm the efficacy of Oct-4 labeling for SSCs. Notably, the frequency of Oct4-positive cells in human testes was low per seminiferous tubule cross-sections and absent in other types of germ cells and sertoli cells, thus Oct4 will be used to effectively isolate and purify human type A spermatogonia (14). 

However, studding on characterization of SSCs by Oct4 specially in human germ cells was very limited (20). So, as presented in our study, Oct4 as specific marker of human GSCs could be applying for isolation and identification of these stem cells. To prove these results, it will be necessary to transplant this subpopulation into sterile nude mice testis and study the seeding of these cells to seminiferous tubules, although the efficiency of the xenotransplantation assay of primate germ cells to immunodeficient mouse testes need to be explored further (14). PLZF, another spermatogonial stem cells marker was studied in our consideration. PLZF as a spermatogonial stem cell specific transcription factor in the testis is required to regulate self-renewal and maintenance of the stem cell pool (24). This transcription factor exerts growth-suppressive activities accompanied by accumulation of cells in the G0/G1 compartment of the cell cycle. Thus, PLZF is thought to regulate directly the epigenetic repression of chromatin domains required for cell differentiation. 

Our studies support the hypothesis that Plzf maintains the undifferentiated state in cells where it is expressed. Costoya *et al* demonstrated that loss of Plzf function shifts the balance between stem cell self-renewal and differentiation toward differentiation at the cost of self-renewal (24). GS cells also have potential clinical value. Patients with malignancies can become infertile following treatment with radiation or chemotherapy, germ stem cells from a testis biopsy could be used to increase stem cell numbers before autologous germ cell transplantation after the end of treatment, thereby protecting fertility. Thus, GS cells will provide new possibilities in biotechnology and medicine (19).
